# Investigation of Effect of Proposed Two-Stage Foam Injection Method and Modified Additive on Workability of Foam Concrete

**DOI:** 10.3390/ma17092024

**Published:** 2024-04-26

**Authors:** Rauan Lukpanov, Duman Dyussembinov, Aliya Altynbekova, Serik Yenkebayev, Adiya Zhumagulova

**Affiliations:** 1 Solid Research Group, LLP, Astana 010008, Kazakhstan; rauan_82@mail.ru (R.L.); dusembinov@mail.ru (D.D.); yenkebayev-serik@mail.ru (S.Y.); 2 Department of Technology of Industrial and Civil Engineering, L.N. Gumilyov Eurasian National University, Astana 010008, Kazakhstan; adiya_kok6e@mail.ru; 3 Department of Construction, L.N. Gumilyov Eurasian National University, Astana 010008, Kazakhstan

**Keywords:** foam concrete, microsilica, post-alcohol bard, blowing agent, X-ray diffraction analysis, microscopic analysis, compressive strength, water absorption

## Abstract

This article presents the results of an investigation of the proposed method and the influence of a modified additive on foam concrete properties. X-ray diffraction analysis showed that the modified additive has a variable mineralogical composition, and the joint use of the components contributes to the synergistic effect, improving the processes of cement hydration. Microscopy of the foam concrete samples showed the presence of microcracks and micropores in samples both with and without the additive. However, the use of the additive significantly reduced their number and size, which indicates an improvement in the structure of the material. The strength values showed that the samples with the additive have high strength. In particular, the strength values of samples of type 3 at different stages of curing exceed those of samples of type 1 by 1.32–1.51 times and samples of type 2 by 1.07–1.10 times. The obtained strength values are 2.82–3.21 MPa for type 1, 3.64–4.04 MPa for type 2, and 4.39–4.84 MPa for type 3, which corresponds to grade D600. The evaluation of water absorption also confirmed the advantages of the proposed method and the additive, significantly reducing the water absorption of the samples and increasing their hydrophobicity. The obtained values of water absorption are 13.8–16.6% for type 1, 13.7–16.1% for type 2, and 9.5–11.2% for type 3.

## 1. Introduction

In recent decades, significant advances have been made in the theory and technology of concrete. Various technical methods have been applied to effectively control the process of structure formation and to produce heavy and light concretes for various purposes with specified properties [1,2,3].

In modern conditions, foam concrete production is evolving with the introduction of new, more effective blowing agents to the market. Special attention is given to the utilization of energy-saving technologies based on the principles of non-autoclaved foam concrete production [4].

The proposed method of non-autoclaved foam concrete production is based on two-stage foam injection. In the first stage, a low-concentration foam agent solution is introduced during the sand–cement mortar preparation process. Then, in the second stage, a highly concentrated foam solution is added during the formation of the cellular concrete structure. This approach promotes a more homogeneous product by enhancing the distribution of foam throughout the material. Additionally, reducing the water–cement ratio during the production stage shortens the setting time, thereby improving the strength properties of the foam concrete [5,6].

Classic foam concrete, like aerated concrete, is characterized by its porous structure, making it a sought-after material in the construction industry. Aerated concrete is created by introducing gas-forming components that, upon interacting with cement, release gas, forming communicating pores. In contrast, foam concrete is produced by adding ready-mixed foam to a mixture of cement and sand, creating a closed system of pores. However, foam concrete manufacturers often face challenges, such as the instability of the mortar structure, shrinkage, the uneven density of the material, and consequently, the unstable strength and thermal conductivity of the products. These disadvantages stem from several factors, with numerous possible reasons. One of the main factors is the high water–cement ratio, which contributes to the material heterogeneity during the setting process [7,8].

To address this issue, a new approach involving the two-stage introduction of foam into the foam concrete production process is proposed. The initial introduction of a low-concentration foam solution into the mixture allows for a more uniform distribution of the foam, while the subsequent addition of a highly concentrated solution promotes a stable and homogeneous material structure. This method reduces the water–cement ratio and improves the strength and thermal conductivity of the products, making it attractive for industrial applications [9].

The development of new approaches to foam concrete production is an important direction in the construction industry today. Disadvantages associated with classical production methods, such as structural inhomogeneity and material shrinkage during the setting process, necessitate the exploration of innovative solutions. One such breakthrough solution has been the modernization of the technological process for foam concrete production. These new technologies have facilitated improvements in the quality of the pore structure and the material density, addressing several challenges. However, the issue of material shrinkage, which impacts the final properties of products, remains pertinent. Despite this challenge, the classical production method persists in construction, with the integration of various additives and enhancements. The ongoing pursuit of novel foam concrete production methods enables the construction industry to enhance the quality of building materials, thereby enhancing the efficiency and durability of building structures [10,11,12,13].

The use of foam concrete in construction offers several advantages, including its high technological ergonomics and excellent thermophysical characteristics. Foam concrete structures exhibit outstanding thermal insulation properties, making them an ideal choice for construction in regions with harsh continental climates and permafrost zones [14,15].

The thermal insulation properties of foam concrete surpass those of other materials, such as expanded clay concrete, clay, and silicate bricks. This is attributed to its unique pore structure, which offers high thermal protection, low material intensity, and environmental friendliness. With the ongoing rise in energy resource costs, the utilization of materials with excellent thermal insulation properties is becoming increasingly pertinent [16,17].

Despite its significant advantages over its counterparts, foam concrete producers often encounter problems such as unstable mortar structure, material shrinkage, and uneven density, which can result in the unstable strength and thermal conductivity of the final product. These drawbacks are typically associated with high water–cement ratios, which can impact the consistency and quality of the concrete. To address these issues, plasticizing admixtures are frequently employed to enhance the characteristics of the concrete mortar. The effective enhancement of concrete quality is achieved through the use of various modifying admixtures to adjust its properties and enhance its performance [18].

This study aimed to evaluate the performance of the proposed method of foam concrete production by two-stage foam injection, as well as the effect of a modified additive on the strength and physical and mechanical properties of the material.

## 2. Materials and Methods

### 2.1. X-ray Diffraction Analysis

An X-ray diffraction analysis of the modified additive was performed to determine its mineralogical composition, as well as the effect of the additive on the phase composition and crystal structure of the cement material to achieve an improvement in the physical and chemical properties of cement, which, in turn, will be necessary to assess the performance of the additive and determine its potential suitability. In this experiment, the test was carried out using a DRON-3 (Burevestnik, St. Petersburg, Russia) type X-ray diffractometer with CuKα radiation. Measurements were carried out under the following conditions: 35 kV; 20 mA; θ–2θ; detector 2 deg/min. The diffractograms of powder samples obtained by the equal-suspensions and artificial-mixtures method were subjected to an X-ray phase analysis on a semi-quantitative basis. As a result, the quantitative ratios of crystalline phases were determined. Samples without and with the additive were prepared for analysis. The samples were ground manually in an agate mortar to a finely ground powder, then dried at 105–110 °C, and sieved through a sieve (No. 008) for the subsequent XRD analysis. The phases were identified by correlation with the corresponding standard X-ray card data (according to ASTM C1365-18 [19]).

### 2.2. Scanning Electron Microscope (SEM)

A microscopic analysis of the structure was performed to qualitatively evaluate the load-bearing skeleton of the foam concrete. The objectives of the visual inspection were to identify skeletal defects—microcracks and micropores in the structure of foam concrete with and without the use of the additive. The quantitative and qualitative evaluation of defects allowed us to evaluate the quality of the material and its serviceability. The microstructure of the materials was studied by using the scanning electron microscopy (SEM) technique. SEM micrographs were obtained with a TM4000 (Hitachi, Tokyo, Japan) scanning electron microscope, with an accelerating voltage of up to 20 KV, magnification of 10× to 25,000×, and resolution for W (3.5 nm).

### 2.3. Compressive Strength of Foam Concrete

A comparison of the strength properties of samples prepared by the proposed method without the additive with those prepared by the classical method was made to assess the performance of the proposed method, indirectly characterizing the uniformity of the pore distribution (taking into account the same quantitative composition of components as the classical foam concrete). A comparison of the strength indices of samples prepared by the proposed method with and without the additive allowed us to evaluate the influence of the additive components on structural improvement based on the strength indices of the pore material skeleton. To determine the compressive strength (according to GOST 25485-2019 [20] and BS EN 12390-Part 3 [21]), cubes (100 × 100 × 100 mm) for each mixture were cast and tested after 7, 14, and 28 days of curing in laboratory conditions using Press Automatic Pilot equipment, with a total compressive load of 500 kN (50 tons).

### 2.4. Water Absorption of Foam Concrete

A comparison of the water absorption of foam concrete allowed us to evaluate the serviceability of foam concrete, primarily related to the service life of the material. The hydrophobicity of the material characterizes its resistance to the destructive effect of water during operation, as well as its increase in frost resistance (taking into account the mechanics of frost resistance tests). Soaking of the samples was carried out to evaluate the water absorption of the sample (according to GOST 17177-94 [22] and ASTM C642-21 [23]). Soaking was carried out until complete water absorption, up to a constant mass (mass change not more than 0.1% during 120 min of observation). Thus, the estimation of water absorption of the samples is described by Equation (1).

(1) $$W_{s}=\frac{W_{m}\times \rho_{d}}{\left(1-\frac{\rho_{d}}{\rho_{s}}\right)\times 100},$$

where $W_{m}$ is the percentage of moisture content of the sample at maximum impregnation relative to the maximum moisture content at full water absorption of the sample, %; $\rho_{d}$ is the density of dried foam concrete, kg/m^3^; and $\rho_{s}$ is the density of the foam concrete skeleton, kg/m^3^.

When assessing the presence of communicating pores, a blue dye was added to the water to enhance visualization and provide greater contrast. After reaching maximum soaking, the samples were dried to a constant mass, with mass variation not exceeding 0.1% during 30 min of observation.

### 2.5. Two-Stage Foam Injection Method for Foam Concrete Production

**Figure 1:**
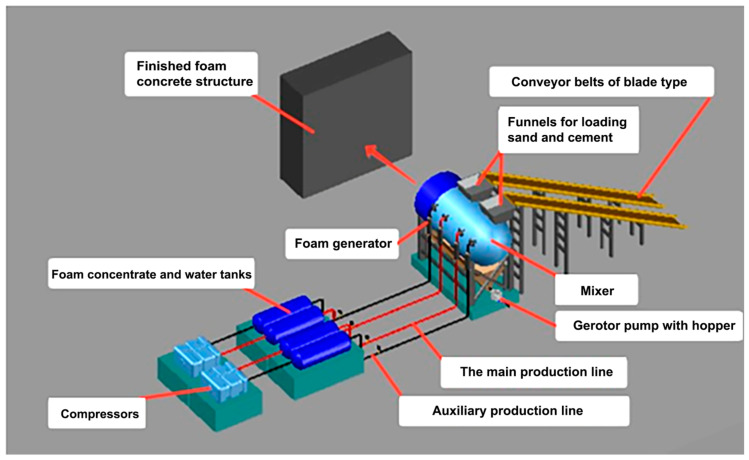
Scheme of foam concrete production by two-stage foam injection [24].

From a technological standpoint, the proposed production method represents a significant departure from previous techniques. It involves a novel two-stage foam injection process, a methodology not previously utilized in foam concrete production (see Figure 1).

The proposed method provides the maximum distribution of the foam concentrate throughout the entire volume of the sample: the primary introduction of a low-concentration foam solution occurs during the preparation stage of the sand–cement mortar, thereby improving its wettability and subsequently reducing the water–cement ratio (thus minimizing foam quenching by water). Subsequently, during the secondary introduction of a highly concentrated foam solution in the manufacturing stage of the cellular concrete structure, the reduction in the water–cement ratio allows for maximal preservation of the initial concentration of the foam concentrate, thereby contributing to the formation of a uniform structure of porous material.

The studies were conducted on foam concrete samples produced using the proposed two-stage foam injection method with the additive (type 3) and without it (type 2). The results were compared with a reference sample produced using classical foam concrete (type 1) production technology. The technological composition of samples produced with each of the compared methods is presented in Table 1.

A total of 6 samples were tested for each of the compared foam concrete sample types to obtain statistical measurement data.

## 3. Results

### 3.1. X-ray Diffraction Analysis of the Modified Additive

**Figure 2:**
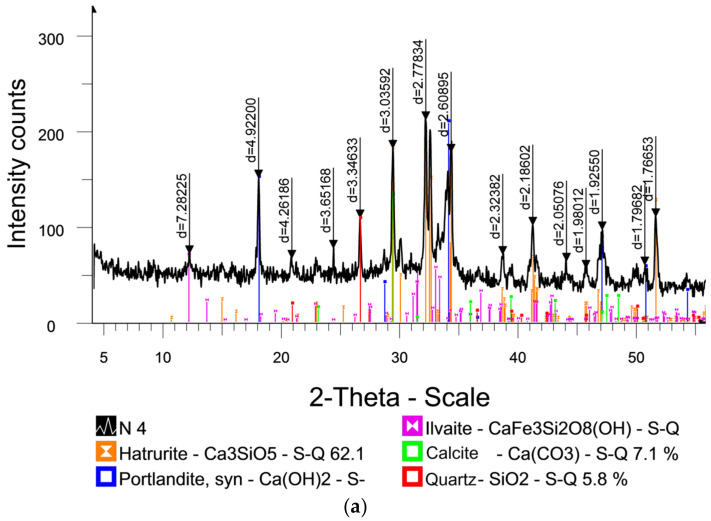

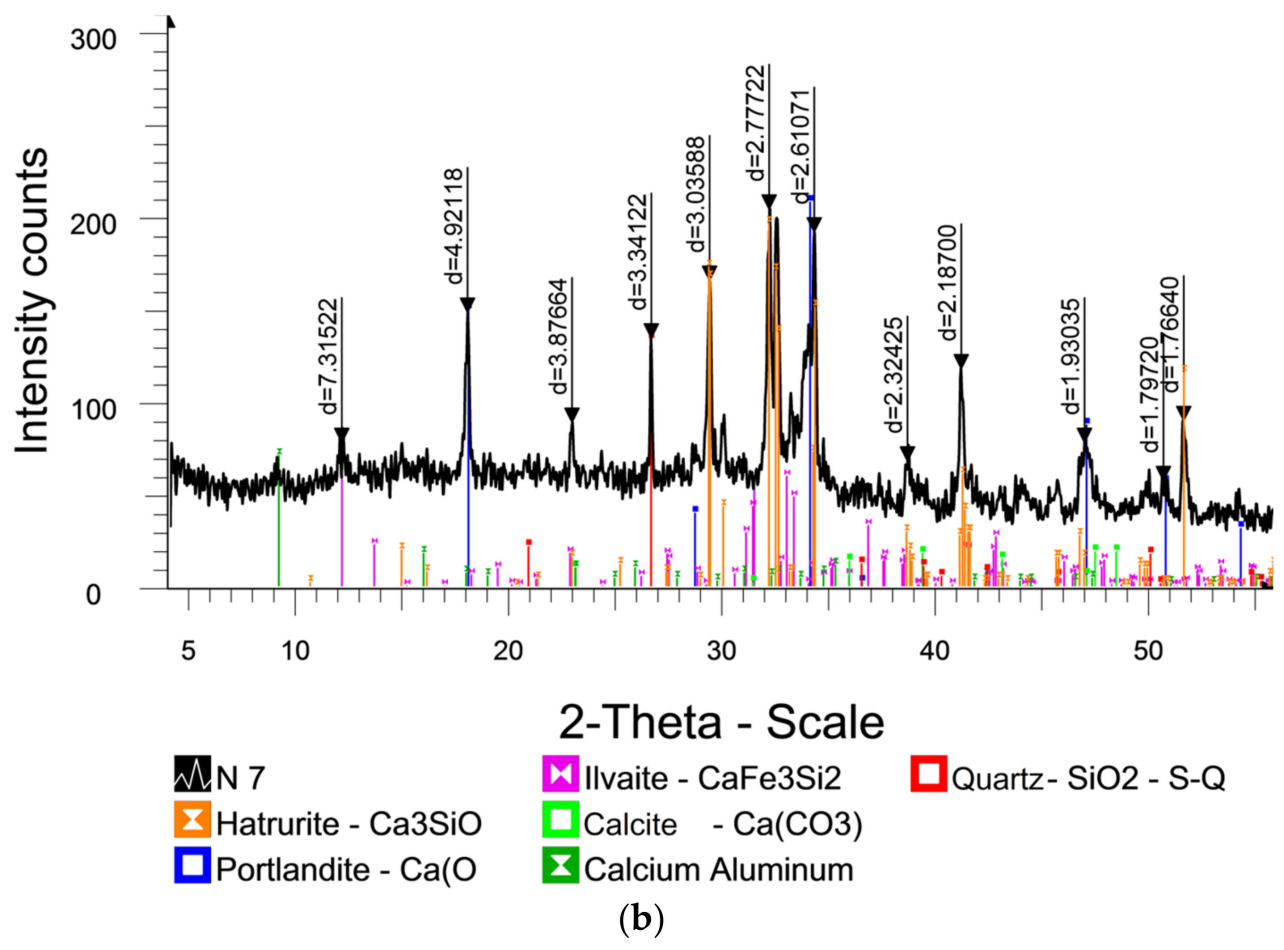
Diffractograms of samples: (**a**) type 2; (**b**) type 3.

Figure 2 shows the diffractogram of the cement binder with and without the modified additive (MA). The elemental composition analysis of samples and their modified forms expressed as weight percentages of oxides by X-ray fluorescence is presented in Table 2 and Table 3 (without MA and with MA, respectively). Each table contains the analytical results for a particular sample and indicates the elemental contents with corresponding concentration values. Table 2 and Table 3 show the chemical composition results, providing information on the compositions of the samples based on the identification of the different mineral phases.

The reference sample (without additive) contained the compounds Ilvaite (CaFe_3_Si_2_O_8_(OH)) with parameter d = 7.28225, 4.92200, 4.26186, and 3.65168; quartz (SiO_2_) with parameter d = 3.34633; calcite (Ca(CO_3_)) with parameter d = 3.03592 and 2.97138; and Hatrurite (Ca_3_SiO_5_) with parameter d = 2.77834, 2.74688, 2.69319, 2.63180, 2.60895, 2.32382, 2.20161, 2.18602, 2.17039, 2.05076, 1.98012, 1.93646, 1.92550, 1.79682, and 1.76653. Ordered by component content, the major phases in Portland cement are Hatrurite (Ca_3_SiO_5_) with 62.1%; Portlandite (Ca(OH)_2_) with 15.7%; Ilvaite (CaFe_3_Si_2_O_8_(OH)) with 9.3%; calcite (Ca(CO_3_)) with 7.1%; and quartz (SiO_2_) with 5.8%. The sample with the additive contained the compounds Ilvaite CaFe_3_Si_2_O_8_(OH) with parameter d = 7.31522, Portlandite Ca(OH)_2_ with parameter d = 4.92118 and 3.87664, quartz SiO_2_ with parameter d = 3.34122, calcite Ca(CO_3_) with parameter d = 3.03588 and 2.97133, and Hatrurite Ca_3_SiO_5_ with parameter d = 2.77722, 2.74802, 2.69303, 2.61071, 2.32425, 2.18700, 1.93035, 1.79720, and 1.76640. Ordered by component content, the major phases in the sample are Hatrurite (Ca_3_SiO_5_) with 56.2%, Portlandite (Ca(OH)_2_) with 15.3%, Ilvaite (CaFe_3_Si_2_O_8_(OH)) with 9.9%, calcite (Ca(CO_3_)) with 5.2%, Calcium Aluminum Silicate (Ca-Al-Si-O) with 6.3%, and quartz (SiO_2_) with 7.1%.

The analysis of the diffractogram of the compared samples revealed the presence of several compounds. In the sample without the additive, the compounds Ilvaite (CaFe_3_Si_2_O_8_(OH)), quartz (SiO_2_), calcite (Ca(CO_3_)), and Hatrurite (Ca_3_SiO_5_) were identified. In samples with the MA, the content of each phase underwent some changes. Additionally, phases like Calcium Aluminum Silicate (Ca-Al-Si-O) were also observed.

Based on the X-ray diffraction analysis data, silicon (Si), calcium (Ca), and oxygen (O) were the predominant elements in the samples.

The analysis showed that the content of calcium hydroxide in the samples with the additive (MA) was lower compared to those without it. This reduction indicates an acceleration in cement hydration due to the decrease in calcium hydroxide (Ca(OH)_2_) content resulting from the hydrolysis of clinker minerals. However, the continued presence of Ca(OH)_2_ (free lime) in concrete can lead to increased internal tension, efflorescence formation, internal corrosion, and loss of strength.

One negative factor affecting foam concrete is the reduction in its physical and mechanical properties during operation and storage. These inconsistencies are often caused by the presence of free lime. The X-ray diffraction analysis revealed a decrease in free lime content with increasing additive content. Based on the obtained results, it can be concluded that increasing the additive content increases SO_3_ and decreases Ca(OH)_2_. During long-term hydration, Ca(OH)_2_ transitions to CaSO_4_, contributing to the primary strength in the initial 3 days before further reacting to form gypsum.

### 3.2. Microscopic Analysis of Foam Concrete Structure

**Figure 3:**
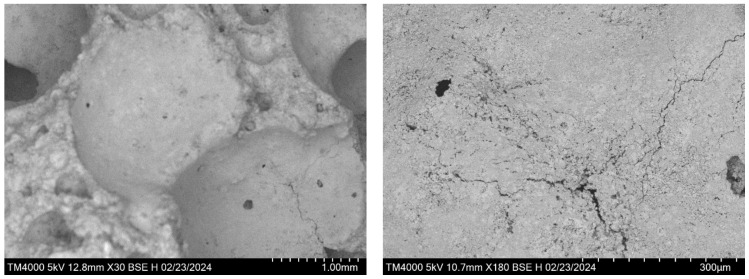
Microphotography of foam concrete samples.

To evaluate the skeletal structure of foam concrete, microphotography of the samples was performed. Comparisons were made between the visualizations of the samples with and without the additive (Figure 3).

As a result of the examination of the material structure, a qualitative evaluation of the load-bearing skeleton of foam concrete was conducted. In samples without the additive, numerous cracks were revealed in the skeleton structure, contrasting with samples with the additive. Visual inspection also identified micropores in the skeletal structure of both untreated and additively treated samples. However, the number of micropores in the samples without the additive was several times higher than in those with the additive. Additionally, the dimensions of the micropores differed: samples with the additive had smaller micropores compared to those without the additive. As expected, the use of the additive improved the structure of foam concrete.

The additive used contains post-alcohol bard, which possesses cement plasticizing properties. When combined with microsilica, it exhibits high moisture adsorption due to its large specific surface area. This characteristic reduces the setting time, allowing the foam concrete mixture to set without damaging shrinkage after the foam introduction. The concurrent application of microsilica and post-alcohol bard reduces the formation of air bubbles on the surface of the non-wetted part of the cement binder. Effective wetting and a reduction in space between the binder and aggregate contribute to the reduction in micropores in the cell wall structure of foam concrete.

### 3.3. Strength Testing of Foam Concrete Samples

**Figure 4:**
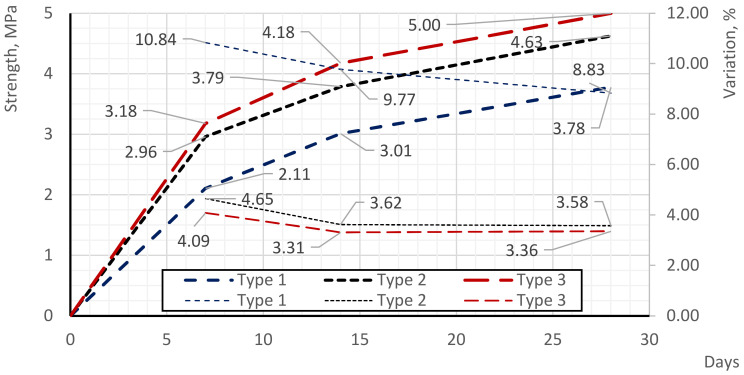
Partial values of compressive strength of samples.

Figure 4 depict plots illustrating the partial variations in the strength of foam concrete samples at different ages, while Figure 4 displays the average values of this relationship for each of the compared methods. It also includes the coefficients of variation of the partial values corresponding to different curing ages.

The strength indices of type 1 samples vary as follows: at 7 days of age, from 1.81 to 2.34 MPa with a coefficient of variation of 10.84%; at 14 days, from 2.78 to 3.19 MPa with a coefficient of variation of 9.87%; and at 28 days, from 3.36 to 4.20 MPa with a coefficient of variation of 8.83%. For type 2 samples, the values are as follows: at 7 days, from 2.82 to 3.21 MPa with a coefficient of variation of 4.65%; at 14 days, from 3.64 to 4.04 MPa with a coefficient of variation of 3.62%; and at 28 days, from 4.39 to 4.84 MPa with a coefficient of variation of 3.58%. Type 1 samples exhibit the lowest average strength values, while type 3 samples demonstrate the highest. The difference in the average values (as well as partial values) of strength indices between type 1 and type 2 samples ranges from 22.41 to 40.61% (at different curing ages), indicating the influence of the production process on strength improvement in type 2 samples compared to type 1. The difference in strength between type 2 and type 3 samples ranges from 7.34 to 10.23% (at different curing ages), highlighting the impact of the modified additive on foam concrete strength. Comparing type 1 and type 3 samples, the strength of type 3 samples is 32.27 to 50.93% higher than that of type 1 samples (depending on the age of curing), underscoring the combined effect of the production process and modified additive on strength enhancement in type 3 samples.

The evaluation of coefficients of variation reveals that type 3 samples have the smallest variation in partial strength values, followed by type 2 samples and then type 1 samples. This indicates greater stability in type 2 and type 3 samples compared to type 1 samples, potentially linked to the uniform distribution of the pore structure.

### 3.4. Testing of Foam Concrete Samples for Water Absorption

**Figure 5:**
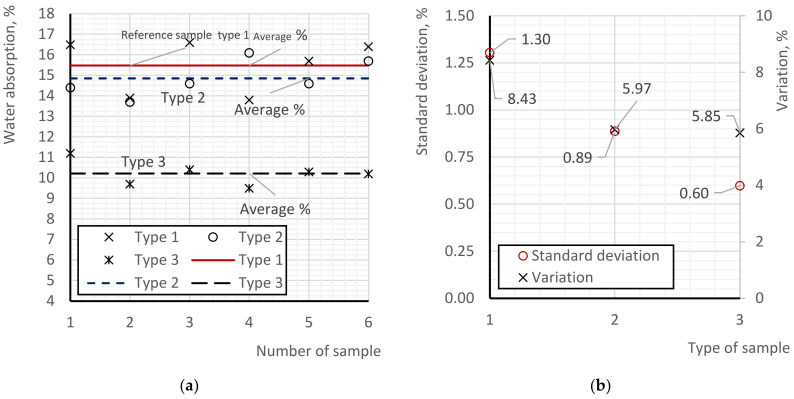
Results of water absorption tests of foam concrete: (**a**) comparison of types, (**b**) statistical processing of data.

The results of the water absorption tests of the samples are presented in Figure 5. Figure 5a displays the partial water absorption values and compares the average water absorption values of foam concrete of comparable types. Figure 5b illustrates the coefficients of variation and quadratic deviations of the partial values.

According to the results obtained, the maximum (worst) water absorption values were found in all type 1 samples. The water absorption values ranged from 13.8 to 16.6%, with an average value of 15.48%. Type 2 samples showed lower water absorption values, ranging from 13.7 to 16.7%. The average value of type 2 samples was 14.85%, which is 4.09% lower than the average water absorption values of type 1 samples. Although the difference is insignificant, the water absorption measurements of type 2 samples relative to type 1 indicate the influence of the two-stage foam injection manufacturing process. The reduction in water absorption can be attributed to the uniformity of the pore structure distribution of foam concrete in type 1 samples compared to type 2. Considering the same density of the samples (i.e., the parity of the ratio of the pore volume to the skeleton volume of type 1 and type 2 samples), we can conclude that the reduction in water absorption is associated with a greater number of communicating pores in type 1 samples compared to type 2, primarily due to the presence of large-sized pores in type 1 samples (mainly in the upper part). The evaluation of the coefficients of variation of type 1 and type 2 samples indirectly confirms the low stability of the pore structure of type 1 samples (from sample to sample). The coefficient of variation of type 2 samples is 30% lower than that of type 1 samples. If we compare type 2 samples to type 1 samples, the coefficient of variation of type 2 is 41% lower. The assumption about the influence of pore structure on water absorption arises from the conditions (considering the same absorption capacity of the foam concrete skeleton), particularly the presence of large pores (the larger the pore, the greater the surface area, and thus the larger the area of contact with water) and the presence of communicating pores (allowing water to penetrate into the internal pores of foam concrete).

The lowest water absorption values were found in type 3 samples, with partial values ranging from 9.5 to 11.2%. The average water absorption values of type 3 samples were 10.22%, which is 34.02% lower than the corresponding values of type 1 samples and 31.20% lower than those of type 2 samples. A significant decrease in the water absorption capacity of type 3 foam concrete samples compared to type 1 and type 2 is observed. In this case, the logical influence of post-alcohol bard (due to its hydrophobic properties), as well as the influence of microsilica (due to the clogging of micropores), is confirmed. Generally, the coefficients of variation of type 2 and type 3 samples are close to each other, being 5.97% and 5.85%, respectively.

**Figure 6:**
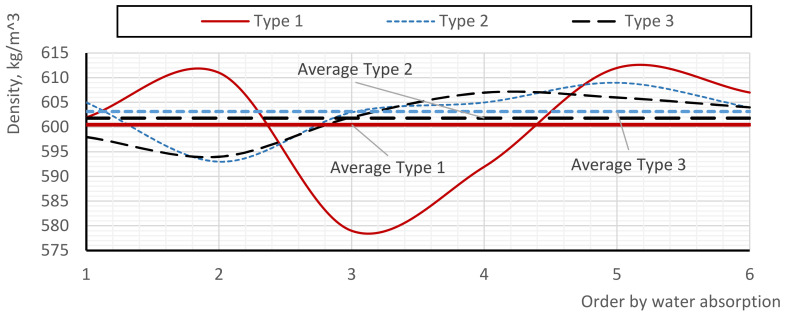
Density deviations from order by moisture content.

Figure 6 shows the plots of the water absorption capacity versus the density of the samples. Theoretically, the smaller the pores (lower density), the lower the water absorption of the sample, which is due to the reduced contact area of the absorbing surface with water.

For a better analysis of the data, the abscissa axis shows the ordinal numbers corresponding to the partial values of individual samples sorted in ascending order of water absorption capacity, while the corresponding density indices are displayed on the ordinate axis. The average density values are also presented in the diagrams. Therefore, the greater the fluctuation of the curves (partial density values) from the straight lines (average values), the more heterogeneous the pore structure. This heterogeneity influences the order of dependence of water absorption on the density and introduces randomness into this regular proportionality.

According to the graph, the maximum deviations of the curve from the mean value are observed in samples of type 1, which also have a greater number of peaks. Interestingly, in samples with both minimum and maximum water absorption, the density is greater than average. Conversely, in samples with average values of water absorption, the density is the lowest, which contradicts the previously suggested pattern. The density fluctuation of type 1 samples varies from −3.6 to 1.9%, corresponding to absolute density values of 579 and 612 kg/m^3^ (with an average of 600 kg/m^3^). The deviations of type 2 and 3 curves have a similar character, with the curve extrema being much smaller than those of type 1. The density fluctuation of type 2 samples varies from −1.6 to 0.9%, corresponding to absolute density values of 593 and 609 kg/m^3^ (with an average of 603 kg/m^3^). Similarly, the density fluctuation of type 3 samples varies from −1.3 to 0.8%, corresponding to absolute density values of 594 and 607 kg/m^3^ (with an average of 602 kg/m^3^). The evaluation of the variation in partial density values also showed the low stability of the pore structure of type 1 foam concrete: the coefficient of variation of type 1 samples is 58–61% higher than the same coefficient of type 2 and 3 samples, while the coefficient of variation of type 2 and 3 samples is 138–157% lower than that of type 1 samples.

**Figure 7:**
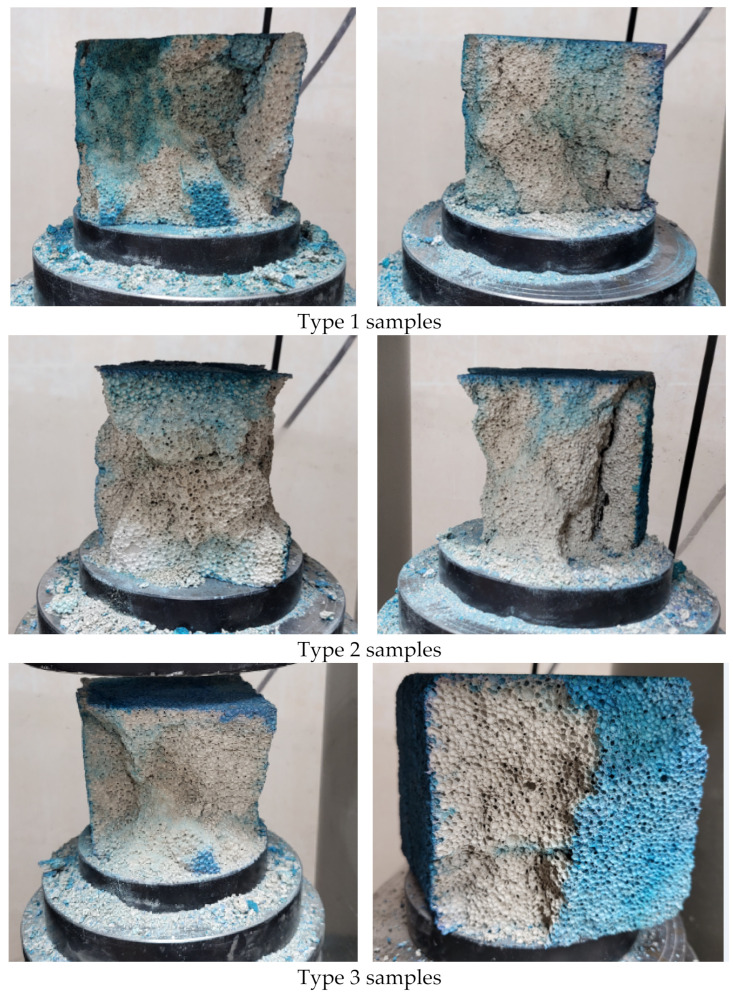
Penetration of pigmented water into the foam concrete structure.

**Figure 8:**
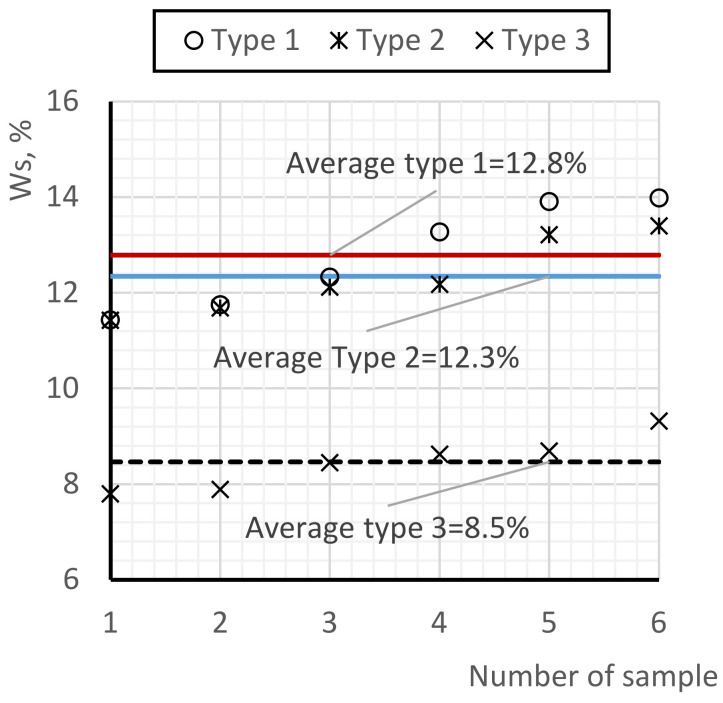
Water absorption of water-soaked samples.

Figure 7 shows the results of the evaluation of water penetration into the pore structure of foam concrete. A qualitative evaluation of pigmented water penetration was performed by visualizing objects; however, for a quantitative evaluation, charts were constructed to characterize the quantitative evaluation of the water penetration capacity. The graphs in Figure 8 show the percentage of moisture content of the maximally impregnated sample relative to the maximum moisture content at full water absorption of the sample.

According to the visual evaluation of the pigmentation of samples, it can be seen that the maximum water penetration is observed in samples of type 1, where the pigmentation area of the internal pore structure of foam concrete is visually superior to the pigmentation areas of samples of types 2 and 3. In type 2 samples, the pigmentation of the pore structure is lower than in type 1 samples, which indicates the influence of the technological process of foam concrete production on the quality of the pore structure. In samples of types 2 and 3, the pigmentation of the pore structure also differs; the samples of type 3 are less pigmented than the samples of type 2. The latter indicates the influence of the modified additive to promote the hydrophobization of the foam concrete skeleton and the clogging of micropores by microsilica.

Quantitatively, type 1 samples exhibit the highest water absorption at 12.8% of the maximum water absorption (when pores are fully filled with water), followed by type 2 samples at 12.3% and type 3 samples at 8.5%.

## 4. Discussion

The results obtained correlate well with similar studies of foam concrete [5,6,9,24,25,26,27], where investigations into the physical and mechanical properties of foam concrete were conducted, often comparing variant compositions. The test results from these studies fall within acceptable limits in comparison with the results obtained in the present study. Any differences in numerical values (within a range of up to 25%) can be attributed to variations in technological compositions and foam concrete production methods.

For instance, the invention «Foaming agent for the manufacture of foam concrete» (No. 7141, p04v 38/10) [28] differs from the proposed method both in composition (consisting of ground sand, sulfanol or sodium salts of alkyl aromatized sulfonic acids PO-1 or PO-6K, aluminum sulfoxide or sodium tripolyphosphate or trisodium phosphate, and cellulose ether) and in production technology, where the use of the blowing agent preserves foam stability while reducing its concentration.

There are other known inventions that propose modernizing the composition of foam concrete components to enhance its quality, such as No. 7140, C04B 38/10, C04B 38/02; No. 15713, C04B 28/04, C04B 38/10, C04B 14/06; KZ A4 24246, C04B 7/38, etc. [29,30,31]. These inventions aim to increase the durability, strength, and resistance of foam concrete through a fundamentally different approach—by developing a new composition of foam concrete.

The proposed method for producing non-autoclaved foam concrete differs fundamentally in both composition and production technology. It involves a two-stage injection of foam: initially, a low-concentration foam solution is introduced during the preparation of the sand–cement mortar, followed by the introduction of a highly concentrated foam solution during the production of the aerated concrete structure.

From the X-ray diffraction analysis, the mineralogical composition of the modified additive, comprising microsilica and post-alcohol bard, was obtained. The evaluation of the mineralogical composition revealed a positive pattern from the combined application of microsilica, resulting in a synergistic effect with the post-alcohol bard. It is known that microsilica enhances the quality of the cement binder, contingent upon the presence of effective plasticizing additives that reduce micropores, thereby improving the contact zone during cement hydration.

The results obtained in this research align with the regulatory standards outlined in GOST 25485-2019 and GOST 17177-94 [20,21,22,23]. The density of the foam concrete obtained, corresponding to grade D600, varies from 579 to 612 kg/m^3^ (type 1), from 593 to 609 kg/m^3^ (type 2), and from 594 to 607 kg/m^3^ (type 3), with a coefficient of variation ranging from 0.9 to 2.1% [20,23].

According to the evaluation of the strength performance of foam concrete, the curves of strength gain over time were obtained, represented by partial and averaged strength gain during the hydration period. Type 3 samples produced by the proposed method of two-stage foam injection with the use of the additive showed the highest strength. At all stages of control measurements and in different curing periods, the strength of type 3 samples exceeded the strength of type 1 samples (standard samples) by 1.32–1.51 times and exceeded the strength of type 2 samples (samples by the method of two-stage foam injection without additive) by 1.07–1.10 times. The evaluation of the coefficients of variation shows that the samples of type 3 have a smaller variation in partial strength values compared to the samples of type 2, and the samples of type 2 have less variation relative to the samples of type 1. These results characterize the greater stability of samples of types 2 and 3 relative to samples of type 1, which may be indirectly related to the uniformity of the pore structure distribution. The strength values obtained for type 1 range from 2.82 to 3.21 MPa, for type 2 from 3.64 to 4.04 MPa, and for type 3 from 4.39 to 4.84 MPa, corresponding to the structural and insulating foam concrete grade D600 [20]. Additionally, the water absorption values obtained for type 1 range from 13.8 to 16.6%, for type 2 from 13.7 to 16.1%, and for type 3 from 9.5 to 11.2%, meeting the permissible humidity values of up to 20% [22].

From the analysis of the water absorption indexes of foam concrete, partial and average values of the water absorption capacity of the compared types of foam concrete were obtained. The results showed a significant decrease in the water absorption capacity of foam concrete samples of type 3 relative to types 1 and 2. The water absorption of type 3 samples is 1.34 times less than that of type 1 and 1.31 times less than that of type 2. In this case, the logical influence of post-alcohol bard (due to hydrophobizing properties) as well as the influence of microsilica (due to clogging of micropores) is confirmed. The evaluation of the water absorption capacity in relation to the density of the samples showed the low stability of the pore structure of foam concrete type 1 in relation to the samples of types 2 and 3, as evidenced by the obtained unevenness of the density distribution of the samples from the increasing order of change in water absorption. The evaluation of the pigmented areas of the samples visually confirmed the influence of the technological process of production and the use of the modified additive on the quality of the pore structure of foam concrete: the area of pigmentation in the samples of type 1 and type 2 visually exceeds the area of the samples of type 3. Quantitatively, the maximum water absorption of type 1 samples is 12.8% of the maximum water absorption (at 100% water filling of the pores), with 12.3% for type 2 samples and 8.5% for type 3 samples.

It is important to note that the proposed technology and technological composition are only applicable for producing foam concrete of grade D600; adjustments to the composition are necessary for other grades of foam concrete. The use of modifiers such as microsilica and post-alcohol bard, as presented in this article, is also applicable to the production of foam concrete by two-stage foam injection. However, further research and an adjustment of the percentage ratio of cement to water are required for producing heavy concrete.

For the practical introduction of foam concrete using the proposed method to the construction market, product approval and the production of a pilot batch under industrial production conditions are necessary steps. Additionally, future research should focus on studying the technological compositions of foam concrete produced by two-stage foam injection intended for thermal insulation (grades D300–D500) and structural (grades D1000–D1200) purposes.

## 5. Conclusions

This study has focused on investigating the effect of the proposed two-stage foam injection method and a modified additive on the workability of foam concrete. The conclusions are as follows:

1. X-ray diffraction analysis studies have shown that the use of a modified additive including microsilica and post-alcohol bard leads to a synergistic effect. This confirms the positive effect of the complex application of these substances on the quality of the cement binder. The studies also indicate changes in the mineralogical and chemical composition of the cement binder, which affect its hydration.
2. Studies on the structure of foam concrete showed the following: the samples without the additive had structural microcracks, while the samples with the additive had significantly fewer such cracks; the number of micropores in the samples without the additive was several times higher than in the samples with the additive, and the size of micropores was also larger in the samples without the additive. Thus, the use of the additive leads to an improvement in the quality of the load-bearing skeleton of foam concrete, which affects its performance as a building material.
3. The strength values showed that the samples with the additive have high strength. In particular, the strength values of samples of type 3 at different stages of curing exceed those of samples of type 1 by 1.32–1.51 times and samples of type 2 by 1.07–1.10 times. The obtained strength values are 2.82–3.21 MPa for type 1, 3.64–4.04 MPa for type 2, and 4.39–4.84 MPa for type 3, which corresponds to grade D600.
4. The evaluation of water absorption also confirmed the advantages of the proposed method and the additive, significantly reducing the water absorption of the samples and increasing their hydrophobicity. The obtained values of water absorption are 13.8–16.6% for type 1, 13.7–16.1% for type 2, and 9.5–11.2% for type 3.

## Figures and Tables

**Table 1:** Technological composition of the compared samples.

Index	Unit	Type 1	Type 2	Type 3
Microsilica	kg	-	-	60
Cement grade M400	kg	320	320	260
Fine sand	kg	220	220	220
Post-alcohol bard	L	-	6	6
Blowing-agent-to-water ratio during initial injection	g:L	1.5:115	0.23:75	0.23:69
Ratio of blowing agent to water during secondary injection	g:L	-	1.27:40	1.27:40

**Table 2:** The chemical composition of the original cement without the MA (type 2).

Statistic	Content, %									
	Na_2_O	MgO	Al_2_O_3_	SiO_2_	SO_3_	K_2_O	CaO	TiO_2_	MnO	FeO
Average	0.12	1.05	3.80	21.60	3.39	0.80	65.18	0.25	0.24	3.56
Std. deviation	0.16	0.08	0.30	0.44	0.08	0.10	0.27	0.13	0.15	0.14
Max.	0.23	1.14	4.07	21.91	3.44	0.90	65.48	0.34	0.42	3.65
Min.	−0.05	1.00	3.47	21.10	3.30	0.72	64.95	0.11	0.12	3.39

**Table 3:** The chemical composition of the sample with the addition of the MA (type 3).

Statistic	Content, %									
	Na_2_O	MgO	Al_2_O_3_	SiO_2_	SO_3_	K_2_O	CaO	TiO_2_	MnO	FeO
Average	0.16	1.13	3.87	19.44	3.77	0.66	66.37	0.29	0.28	4.03
Std. deviation	0.02	0.22	0.34	0.38	0.12	0.13	1.20	0.11	0.09	0.28
Max.	0.18	1.31	4.26	19.81	3.85	0.76	67.54	0.37	0.38	4.33
Min.	0.14	0.89	3.60	19.06	3.63	0.52	65.14	0.17	0.20	3.76

## Data Availability

Data are contained within the article.
